# miR-124 and miR-142 enhance cisplatin sensitivity of non-small cell lung cancer cells through repressing autophagy *via* directly targeting SIRT1

**DOI:** 10.1039/c8ra09914f

**Published:** 2019-02-12

**Authors:** Xiang Song, Fanyi Kong, Zhenfeng Zong, Mingming Ren, Qingjun Meng, Yanguang Li, Zhen Sun

**Affiliations:** Department of Thoracic Surgery, Cangzhou Central Hospital No. 16, Xinhua West Road, Canal District Cangzhou 061000 China songxiangsx1@163.com +86-0317-2075527

## Abstract

*Background*: Drug resistance is a major obstacle in the treatment of non-small cell lung cancer (NSCLC). Recently, miRNAs are reported to be involved in the drug resistance of NSCLC. The roles of miR-124 and miR-142 in the multidrug resistance of NSCLC cells have been reported. However, the underlying mechanism by which miR-124 and miR-142 regulate resistance to cisplatin (CDDP) remains unknown. *Methods*: The expressions of miR-124, miR-142 and sirtuin 1 (SIRT1) in CDDP-sensitive and CDDP-resistant NSCLC tissues and cells were detected by qRT-PCR and western blot. IC50 value and cell proliferation were determined by MTT assay. Apoptosis was assessed by flow cytometry analysis. Autophagy was evaluated by western blot analysis of the protein levels of LC3-I, LC3-II and p62, and FITC-LC3 punctate formation assay. The interaction between miR-124 or miR-142 and SIRT1 was determined by luciferase reporter, RNA immunoprecipitation (RIP) and western blot assays. A tumor xenograft was performed to further validate the role of miR-124 and miR-142 in the sensitivity of CDDP-resistant NSCLC to cisplatin. *Results*: miR-124 and miR-142 were downregulated, while SIRT1 was upregulated in CDDP-resistant NSCLC tissues and cells compared to CDDP-sensitive groups. Functionally, overexpression of miR-124 and miR-142 or SIRT1 silencing enhanced the CDDP sensitivity of H1299/CDDP cells *via* suppressing autophagy, as evidenced by the reduced LC3-II/LC3-I radio, elevated p62 protein, and suppressed FITC-LC3 punctate formation in H1299/CDDP cells. miR-124 and miR-142 were demonstrated to co-target SIRT1. Re-expression of SIRT1 overturned miR-124 and miR-142-mediated chemosensitivity in H1299/CDDP cells *via* triggering autophagy. *Conclusion*: miR-124 and miR-142 enhance the cytotoxic effect of CDDP through repressing autophagy *via* targeting SIRT1 in CDDP-resistant NSCLC cells.

## Abstract 2

Drug resistance is a major obstacle in the treatment of non-small cell lung cancer (NSCLC).

## Graphical abstract

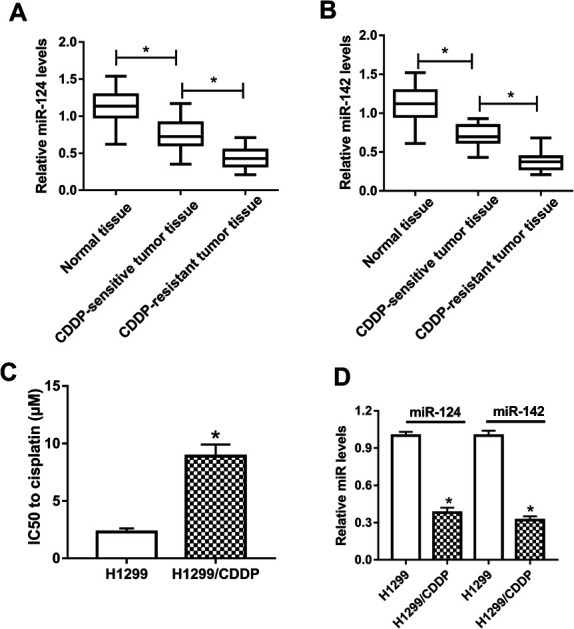

## Introduction

Lung cancer, one of the most commonly diagnosed malignancies worldwide, is the leading cause of cancer-associated morbidity and mortality among both men and women.^1^ Non-small cell lung cancer (NSCLC), the most common form of lung cancer, accounts for approximately 85% of all lung cancer cases with an overall 5 year survival rate as low as 11%.^2,3^ Approximately two-thirds of NSCLC patients are diagnosed at an advanced stage, which makes them candidates for systemic chemotherapy.^4^ Currently, platinum-based chemotherapy is considered as the mainstay of adjuvant chemotherapy for NSCLC patients following surgical resection. As a platinum-based compound, cisplatin (CDDP) is a widely used first-line chemotherapeutic agent for the clinical treatment of advanced NSCLC.^5^ Nevertheless, its therapeutic efficacy is significantly reduced by the acquisition and development of resistance to anti-cancer drugs, leading to chemotherapeutic failure and tumor relapse.^6^ Therefore, further investigations on the molecular mechanism of CDDP resistance and development of novel therapeutic strategies for NSCLC may be advantageous.

MicroRNAs (miRNAs), a class of small, endogenous, non-coding RNA molecules with 19–24 nucleotides, negatively regulate gene expression in a sequence-specific manner by base-pairing with the 3′-untranslated region (3′-UTR) of target mRNA, leading to mRNA degradation and translational repression.^7^ Functionally, there is striking evidence that miRNAs play crucial roles in a variety of critical cellular processes, such as cell differentiation, proliferation, apoptosis, metastasis and angiogenesis.^8^ Recently, increasing studies have suggested that aberrant expression of specific miRNAs is closely implicated in the resistance to chemotherapeutic agents in multiple cancers, particularly in NSCLC.^9,10^ Specifically, miR-142 and miR-124 have been reported to be frequently downregulated and are generally considered as tumor suppressors in several types of malignant tumors including NSCLC.^11–14^ Also, recent studies have identified miR-124 as a novel diagnostic and prognostic biomarker for NSCLC and found that miR-142 was associated with the poor clinical outcome of NSCLC.^15,16^ More notably, it was documented that miR-124 overexpression sensitized gefitinib-resistant NSCLC cells to gefitinib.^17^ Meanwhile, miR-142-3p overexpression enhanced CDDP sensitivity of NSCLC cells through inhibiting anti-cancer drug-induced autophagy.^18^ However, the biological roles and underlying mechanism of miR-124 and miR-142 in CDDP resistance remains unknown.

In our study, we explored the expressions of miR-142 and miR-124 in CDDP-resistant NSCLC tissues and cells. Moreover, we further investigated the effects of miR-142 and miR-124 on the chemosensitivity of CDDP-resistant NSCLC cells and their underlying mechanism.

## Materials and methods

### Patient tissue samples

A total of 36 freshly resected NSCLC tissues and matched adjacent normal tissues were obtained from advanced patients who underwent surgery at Cangzhou Central Hospital and received CDDP-based chemotherapy between January 2016 and December 2017. All excised tissues were promptly snap frozen in liquid nitrogen and preserved at −80 °C for further analysis. These samples, classified histologically by two professional pathologists, were categorized into three groups: normal group (*n* = 36), CDDP-sensitive group (*n* = 18; complete response or partial response) and CDDP-resistant group (*n* = 18; stable disease or progressive disease) according to the Response Evaluation Criteria in Solid Tumors (RECIST). This protocol has been approved by the Ethics Committees of Cangzhou Central Hospital, and the research was performed in accordance with the provisions of Helsinki Declaration revised in 2008, as well as the International Ethical Guidelines for Biomedical Research Involving Human Subjects revised by CIOMS in 2002. Written informed consents were obtained from all participants prior this research.

### Cell culture and transfection

CDDP-resistant cell line H1299 (H1299/CDDP) and the corresponding parental cells H1299 were purchased from the American Type Culture Collection (ATCC, Manassas, VA, USA). All cells were cultured in RPMI-1640 medium (Thermo Fisher Scientific, Inc., Waltham, MA, USA) supplemented with 10% fetal bovine serum (FBS; Gibco, Grand Island, NY, USA), and 1% penicillin/streptomycin at 37 °C in a humidified atmosphere containing 5% CO_2_. To maintain the drug-resistant phenotype, CDDP (Sigma, St. Louis, MO, USA) was added to the culture media at a final concentration of 1 μg ml^−1^ for H1299/CDDP cells.

miR-124 mimics (miR-124) and its matched negative control (miR-NC), miR-142 mimics (miR-142) and its corresponding negative control (miR-nc), small interfering RNA (si-RNA) against sirtuin 1 (SIRT1) (si-SIRT1) and control siRNA (si-NC) were purchased from RiboBio (Guangzhou, China). To overexpress SIRT1, the full-length of SITR1 was synthesized and inserted into pcDNA3.1-overexpressing vector (Invitrogen, Carlsbad, CA, USA) to produce pcDNA-SIRT1 (SIRT1), with pcDNA3.1 empty control (pcDNA) as a negative control. H1299/CDDP cells were seeded into 6-well plates at a density of 2 × 10^5^ cells per well and transfected with these above oligonucleotides or plasmids using Lipofectamine 2000 (Invitrogen) when cells were grown to 70–80% confluence.

### Quantitative real-time PCR (qRT-PCR)

Total RNA was isolated from collected tissues or cultured cells using Trizol reagent according to the manufacturer's instruction (Invitrogen) and quantified by Nanodrop 2000 (Thermo Fisher Scientific, Inc.). cDNA for miR-124 and miR-142 was synthesized using the TaqMan MicroRNA Reverse Transcription kit (Thermo Fisher Scientific, Inc.) and miR-124 and miR-142 expressions were evaluated using TaqMan assay kits (Applied Biosystems, Foster City, California, USA), with U6 small nuclear RNA (snRNA) as an endogenous control. To quantity SIRT1 mRNA, 1 μg RNA samples were reversely transcribed into cDNA mRNA using a PrimeScript™ RT-PCR Kit (Takara, Dalian, China) and qPCR was performed using SYBR Green quantitative PCR SuperMix (Invitrogen), with GAPDH as an internal control. PCR reactions were conducted on an ABI 7500 Real-Time PCR system (Applied Biosystems). The relative gene expression was calculated by the 2^−ΔΔ*C*_t_^ method.^19^

### Cell cytotoxic and proliferation assays

For cytotoxic analysis, H1299 and H1299/CDDP cells treated with varying doses of cisplatin (0–64 μg ml^−1^) were seeded into 96-well plates (5 × 10^3^ cells per well). At 48 h after CDDP administration, 10 μl MTT solution (Solarbio, Beijing, China) was added into the medium and incubated for another 2 h, followed by the introduction of DMSO to dissolve formazan. The absorbance at a wavelength of 490 nm was evaluated on a Microplate Reader (Thermo Fisher). Cell growth inhibition rate (IR) was calculated following the formula: IR = (1 − OD values of control group/OD values of drug group) × 100%, followed by the calculation of 50% inhibitory concentration (IC50) value. For cell proliferation analysis, transfected H1299/CDDP cells were collected at 24 h after transfection and seeded into 96-well plates at a density of 5 × 10^3^ cells per well. Cells were incubated at 37 °C and cell proliferation was determined at 0 h, 24 h, 48 h, and 72 h after incubation as described above. The absorbance at 490 nm was determined on a Microplate Reader (Thermo Fisher).

### Cell apoptosis by flow cytometry

Cell apoptosis was examined using the Annexin V-fluorescein isothiocyanate (FITC) Apoptosis Detection kit (KeyGen Biotech, Nanjing, China) by flow cytometry analysis. Briefly, H1299/CDDP cells were seeded into 6-well plates (1 × 10^5^ cells per well) and transfected with miR-124, miR-142, si-SIRT1, combined with pcDNA or SIRT1, or negative controls. About 48 h after transfection, cells were collected and washed with PBS, resuspended in 200 μl 1× binding buffer, and stained with 10 μl Annexin V-FITC and 5 μl propidium iodide (PI) for 15 min in the dark. The percentage of apoptotic cells was calculated by a flow cytometer (FACSCalibur; BD Biosciences, San Jose, CA, USA).

### Western blot

Total protein lysates were extracted from cultured cells using RIPA buffer (Beyotime, Shanghai, China) in the presence of phosphatase inhibitor cocktail and protease inhibitor cocktail (both Hoffman-La Roche Ltd., Basel, Switzerland). Equal amount of protein extracts was then loaded on 10% sodium dodecyl sulfate-polyacrylamide gel electrophoresis (SDS-PAGE), and transferred onto polyvinylidene fluoride (PVDF) membranes (Millipore, Billerica, MA, USA). After blocked with 5% non-fat milk in Tris-buffered saline with Tween-20 (TBST) for 2 h at 37 °C, the membranes were incubated overnight at 4 °C with the primary antibodies against LC3 (LC3-I and LC3-II) (1 : 1000 dilution, Abcam, Cambridge, UK), p62 (1 : 2000, Cell Signaling Technology, Danvers, MA, USA), SIRT1 (1 : 500, Cell Signaling Technology) and β-actin (1 : 2000, Cell Signaling Technology). After washing, the membranes were further incubated with horseradish peroxidase (HRP)-conjugated secondary antibody (1 : 2000 dilution; Santa Cruz Biotechnology, Santa Cruz, CA, USA) for 1 h at room temperature. Finally, the protein bands were visualized by an enhanced chemiluminescent substrate (Pierce Biotechnology, Rockford, IL, USA).

### FITC-LC3 punctate formation assay

H1299/CDDP cells (5 × 10^4^) were cultured on Polysine microscope adhesion slides (Thermo Fisher) at 37 °C, 5% CO_2_ for 24 h. Then, cells on the slides were fixed in 4% paraformaldehyde and blocked with 2% BSA containing 0.1% Triton-X 100. After incubated with primary antibodies against LC3 overnight at 4 °C, cells were further incubated with FITC-conjugated secondary antibody. Afterwards, cells were washed and stained with 4′,6-diamidino-2-phenylindole (DAPI) (Solarbio), imaged with a confocal laser scanning microscopy.

### Luciferase reporter assay

The entire fragments of SIRT1 3′UTR carrying the putative wild-type (WT) or mutated (MUT) miR-124 or miR-142 binding sites were amplified and inserted into the downstream of psi-CHECK™-2 basic luciferase reporter plasmid (Promega, Madison, WI, USA) using the *Xho* I and *Not* I sites, namely SIRT1-WT and SIRT1-MUT. H1299/CDDP cells (2 × 10^5^ cells per well) were seeded into 24-well plates and co-transfected with 0.2 μg recombinant luciferase reporter vectors and 50 nM miR-124, miR-142, or corresponding controls using Lipofectamine 2000 (Invitrogen). Following 48 h of transfection, the cells were harvested and luciferase activity was measured with Dual Luciferase Reporter Assay System (Promega).

### RNA immunoprecipitation (RIP)

RIP analysis was carried out in H1299/CDDP cells by using Magna RIP RNA-Binding Protein Immunoprecipitation Kit (Millipore). In brief, H1299/CDDP cells were lysed in RIP buffer and incubated with Protein A/G magnetic beads bounded with primary antibodies against Ago2 (Abcam) or IgG (Abcam). After removal of the protein and DNA in immunoprecipitant complex, the enrichment levels of miR-124, miR-142, and SIRT1 were measured by qRT-PCR.

### Tumor xenograft

This study was performed according to the Guidelines for Care and Use of Laboratory Animal with the approval of Ethics Committee of Animal Research of Cangzhou Central Hospital. Male BALB/c nude mice (6 week-old) were purchased from Vital River Laboratory Animal Technology (Beijing, China) and housed in specific pathogen-free condition with a 12 h light/dark cycle. 6 × 10^6^ of H1299/DDP cells were transfected with miR-NC, miR-124, or miR-142 and then subcutaneously injected into the nude mice. When tumor volume reached about 60 mm^3^, mice were treated with 10 mg kg^−1^ of CDDP once per weeks, with untreated group as a blank control. Tumors were measured every seven days and volume was calculated following the formula: volume (mm^3^) = (length × width^2^)/2. At the end time of implantation, tumors were harvested for weighting and expression analysis.

### Statistical analysis

All quantitative data were expressed as the mean ± standard deviation (SD) from three independent experiments. Statistical difference between groups were assessed with Student's *t*-test or one-way analysis of variance (ANOVA) using GraphPad Prism 5 (GraphPad, San Diego, CA, USA). *P* values less than 0.05 were regarded as statistically significant.

## Results

### miR-124 and miR-142 were significantly downregulated in CDDP-resistant NSCLC

**Fig. 1:**
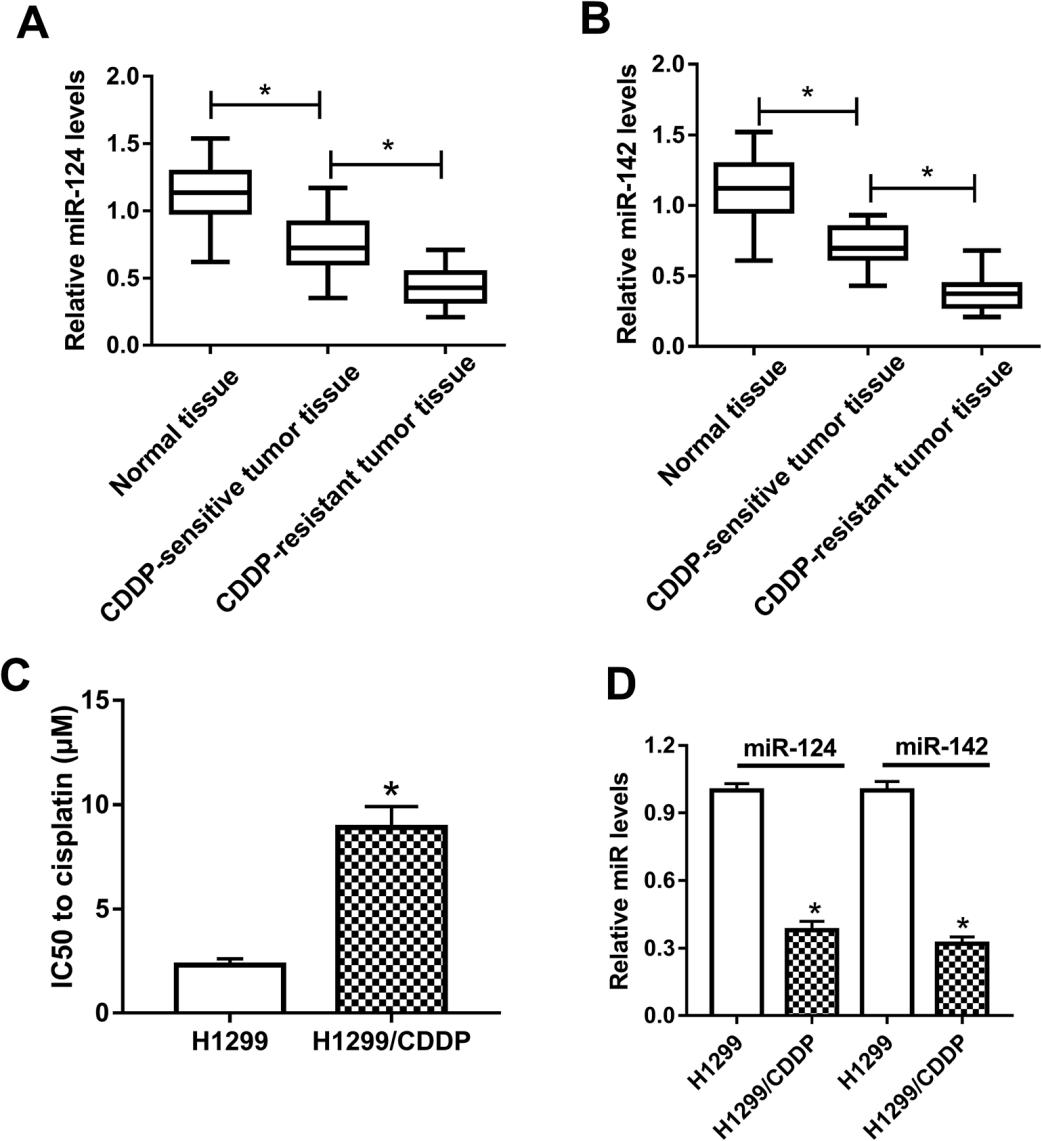
The expressions of miR-124 and miR-142 in CDDP-sensitive and CDDP-resistant NSCLC. (A and B) qRT-PCR analysis was performed to determine the expressions of miR-124 and miR-142 in adjacent normal tissues, CDDP-sensitive and CDDP-resistant NSCLC patients. (C) MTT assay was performed to determine the IC50 value of CDDP in H1299 and H1299/CDDP cells. (D) The enrichments of miR-124 and miR-142 in H1299 and H1299/CDDP cells. *P < 0.05.

To clarify the association between miR-124, miR-142 and CDDP resistance in NSCLC cells, we initially measured the expressions of miR-124 and miR-142 in CDDP-sensitive and CDDP-resistant NSCLC patient tissues. The results displayed that miR-124 and miR-142 expressions were both aberrantly downregulated in CDDP-sensitive and CDDP-resistant NSCLC tumor tissues as compared with adjacent normal tissues, especially in CDDP-resistant NSCLC tumor tissues (Fig. 1A and B). To further demonstrate whether miR-124 and miR-142 was involved in drug sensitivity, CDDP-tolerant NSCLC cell lines H1299/CDDP were obtained and the cytotoxicity of H1299 and H1299/CDDP cells to CDDP was evaluated by IC50 value using MTT assay. Results showed that IC50 values of CDDP were markedly higher in H1299/CDDP cells than in H1299 cells (Fig. 1C). Next, qRT-PCR assay further revealed that miR-124 and miR-142 expression in H1299/CDDP cells was strikingly higher than in H1299 cells (Fig. 1D). These findings indicated that miR-124 and miR-142 might be involved in the CDDP resistance of NSCLC.

### miR-124 and miR-142 enhanced the cisplatin sensitivity of CDDP-resistant NSCLC cells *via* modulation of autophagy

**Fig. 2:**
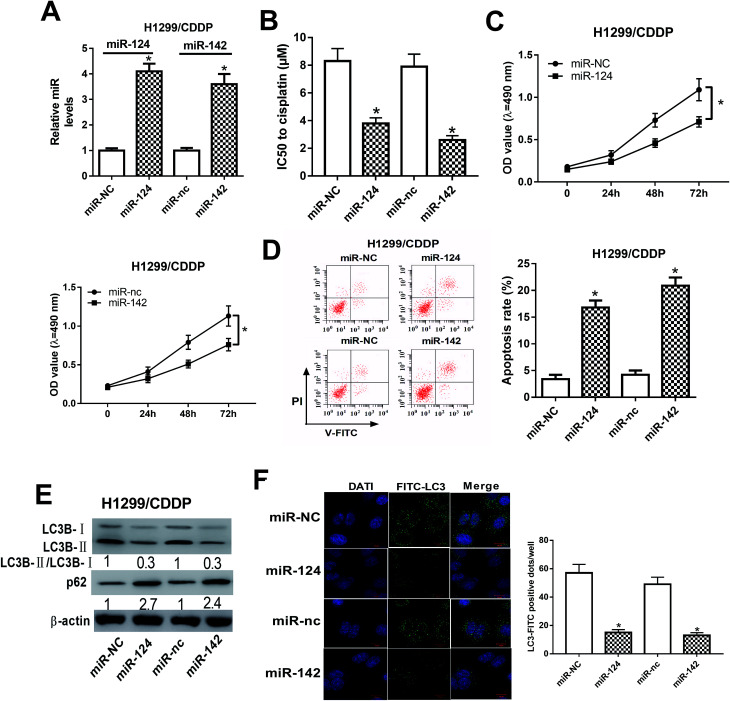
The effects of miR-124 or miR-142 on the chemosensitivity of CDDP-resistant NSCLC cells. H1299/CDDP cells were transfected with miR-NC, miR-124, miR-nc, or miR-142. (A) The expressions of miR-124 and miR-142 in H1299 and H1299/CDDP cells were measured by qRT-PCR. (B–D) MTT and flow cytometry assays were performed to determine IC50 value, cell proliferation, and apoptosis in H1299/CDDP cells. (E and F) Western blot and FITC-LC3 punctate formation were carried out to analyze cell autophagy. *P < 0.05.

To address the roles of miR-124 and miR-142 in CDDP resistance in NSCLC cells, we overexpressed miR-124 and miR-142 in H1299/CDDP cells by transfecting with miR-124 or miR-142 mimics, and qRT-PCR assay confirmed the high transfection efficiency (Fig. 2A). In addition, MTT and flow cytometry analyses implicated that overexpression of miR-124 or miR-142 decreased the IC50 value (Fig. 2B) and inhibited cell proliferation (Fig. 2C), while promoted apoptosis (Fig. 2D) in H1299/CDDP cells. LC3 is one of the biomarkers of autophagy. At the beginning of autophagy, cytosolic LC3-I is converted into membrane-bound LC3-II, which is an essential step in autophagosome formation. Thereby, the abundance of LC3-II is correlated with the number of autophagosomes.^20^ In the following study, we analyzed the effects of miR-124 and miR-142 on autophagy in H1299/CDDP cells by western blot and FITC-LC3 punctate formation analyses. As shown in Fig. 2E, the ratio of LC3-II/LC3-I was dramatically decreased while the protein level of p62, an index of autophagic degradation, was notably increased in miR-124 or miR-142-overexpressed H1299/CDDP cells. Consistently, FITC-LC3 analysis showed that FITC-LC3 positive dots in H1299/CDDP cells transfected with miR-124 or miR-142 were markedly declined compared with matched controls (Fig. 2F), hinting the inhibitory effects of miR-124 and miR-142 on cell autophagy. Together, these data suggested that miR-124 and miR-142 enhanced the chemosensitivity of CDDP-resistant NSCLC cells to cisplatin *via* suppressing autophagy.

### SIRT1 was a direct target of miR-124 and miR-142 in CDDP-resistant NSCLC cells

**Fig. 3:**
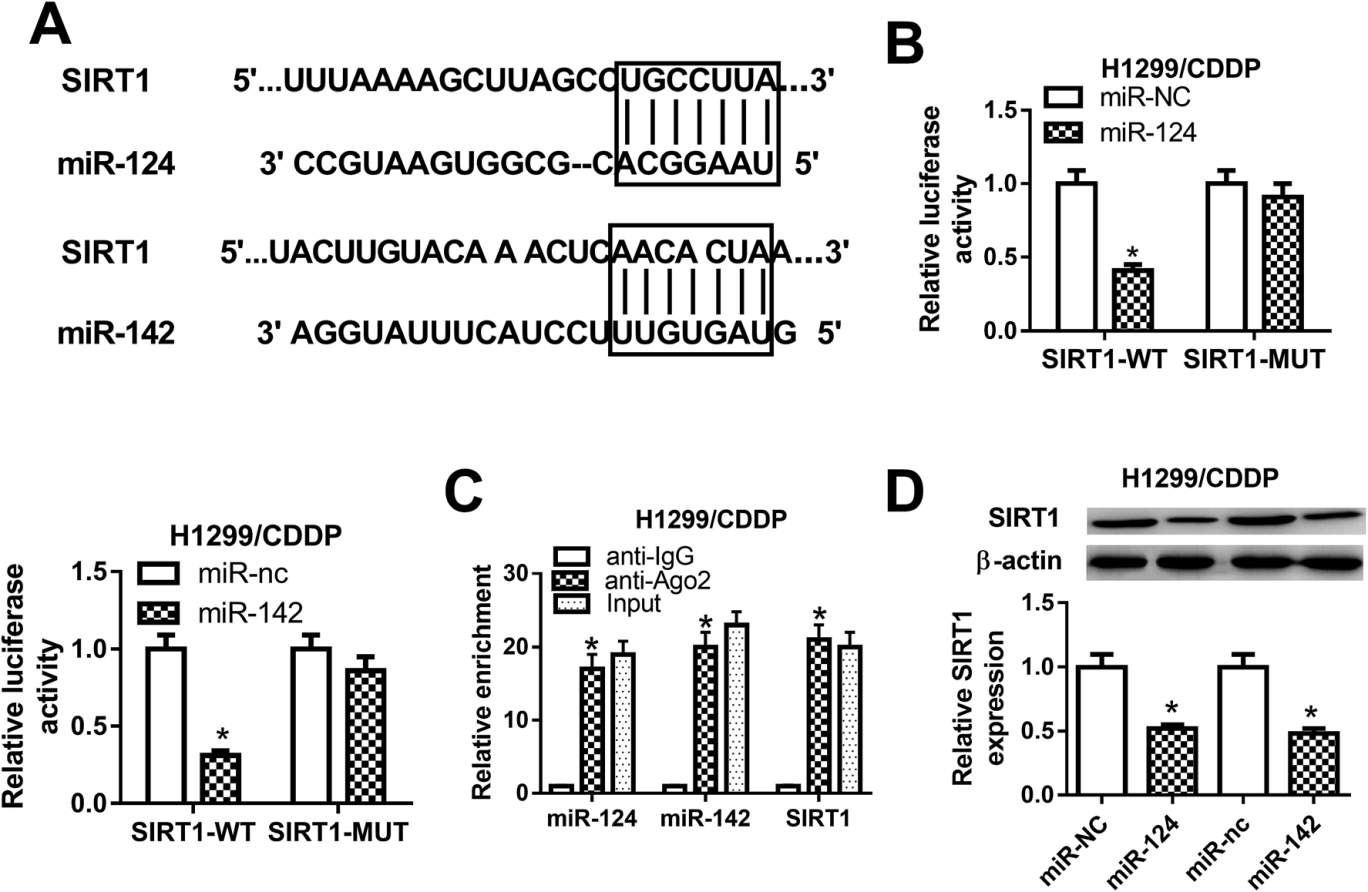
The interaction between miR-124, miR-142 and SIRT1 in CDDP-resistant NSCLC cells. (A) The predicted miR-124 or miR-142 binding sites in the 3′UTR of SIRT1. (B) After H1299/CDDP cells were cotransfected with miR-124, miR-142, or respective controls and luciferase reporter plasmids carrying the wild-type or mutated 3′UTR of SIRT1, luciferase reporter assay was performed to detect luciferase activity. (C) The enrichments of miR-124, miR-142, and SIRT1 were detected by RIP assay. (D) The protein level of SIRT1 in H1299/CDDP cells transfected with miR-124, miR-142, or respective control was evaluated by western blot. *P < 0.05.

To explore the molecular mechanism by which miR-124 and miR-142 mediate CDDP resistance in NSCLC cells, online databases TargetScan were used to predict the potential targets of miR-124 and miR-142. As shown in Fig. 3A, SIRT1 was predicted to be a candidate target of miR-124 and miR-142. To validate our hypothesis, luciferase reporter containing the wild-type 3′UTR of SIRT1 and its corresponding mutant counterparts were constructed and cotransfected with miR-124, miR-142, or matched controls in H1299/CDDP cells. The results from luciferase reporter assay implied that ectopic expression of miR-124 or miR-142 triggered a substantial reduction of luciferase activity of SIRT1-WT in H1299/CDDP cells, but had no significant effect on the luciferase activity of SIRT1-MUT (Fig. 3B). Furthermore, RIP assay was performed using Ago2 antibody to further explore whether miR-124 and miR-142 have a chance to interact with SIRT1. Results showed that miR-124, miR-142, and SIRT1 could be highly enriched by Ago2 antibody in H1299/CDDP cells (Fig. 3C), implying the true interaction between miR-124 or miR-142 and SIRT1. Next, western blot was performed to detect the protein level of SIRT1 in H1299/CDDP cells transfected with miR-124, miR-142, or corresponding controls. The results demonstrated that SIRT1 level was effectively restrained in H1299/CDDP cells following miR-124 or miR-142 overexpression (Fig. 3D). These results demonstrated that SIRT1 was a target of miR-124 and miR-142 in CDDP-resistant NSCLC cells.

### SIRT1 silencing enhanced the cisplatin sensitivity of CDDP-resistant NSCLC cells *via* impeding autophagy

**Fig. 4:**
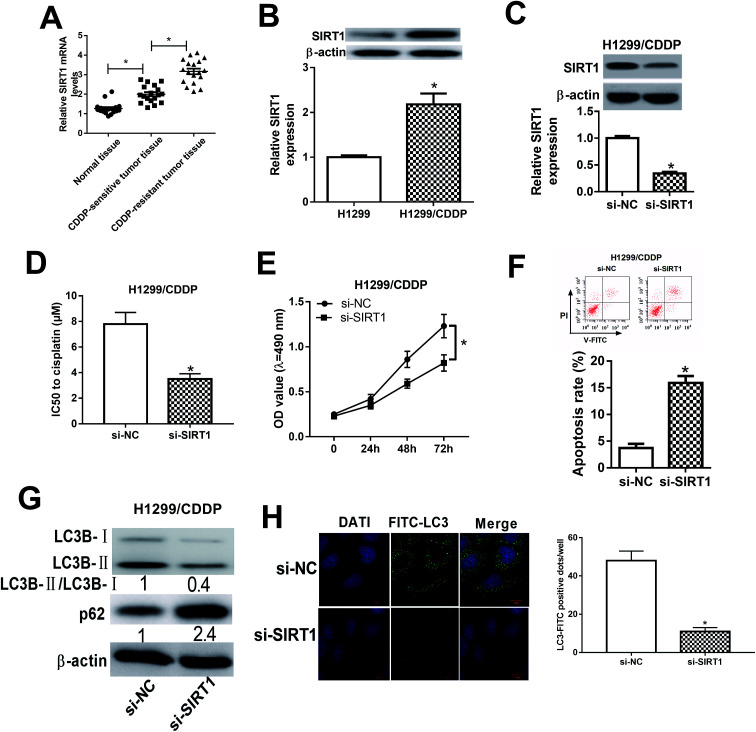
The effects of SIRT1 silencing on the chemoresistance of CDDP-resistant NSCLC cells. (A) The mRNA expression of SIRT1 in CDDP-sensitive and CDDP-resistant NSCLC patients was detected by qRT-PCR. (B) The protein level of SIRT1 in H1299 or H1299/CDDP cells was measured by western blot. (C) The protein level of SIRT1 in H1299/CDDP cells transfected with si-SIRT1 or si-NC was examined by western blot. (D–F) H1299/CDDP cells were transfected with si-NC or si-SIRT1. MTT and flow cytometry assays were carried out to determine IC50 value, cell proliferation, and apoptosis. (G and H) Western blot and FITC-LC3 punctate formation assays were performed to assess cell autophagy. *P < 0.05.

We initially detected the mRNA expression of SIRT1 in CDDP-sensitive and CDDP-resistant NSCLC tumor tissues by qRT-PCR and the results showed that SIRT1 mRNA expression was robustly upregulated in CDDP-sensitive tumor tissues in contrast to adjacent normal tissues (Fig. 4A). Moreover, SIRT1 mRNA expression was distinctly higher in CDDP-resistant NSCLC tissues than that in CDDP-sensitive tissues (Fig. 4A). Meanwhile, we also detected the protein level of SIRT1 in H1299 and H1299/CDDP cells by western blot and discovered that SIRT1 level was highly expressed in H1299/CDDP cells relative to H1299 cells (Fig. 4B). To determine the effects of SIRT1 on CDDP resistance, loss-of-function approaches were performed in H1299/CDDP cells by transfecting with si-SIRT1 or si-NC. Western blot confirmed that si-SIRT1-transfected H1299/CDDP cells showed a significant decrease of SIRT1 level compared with si-NC-introduced cells (Fig. 4C). Functionally, MTT and flow cytometry analysis showed that the IC50 value (Fig. 4D) and cell proliferation (Fig. 4E) was suppressed, and apoptosis (Fig. 4F) was prominently promoted in H1299/CDDP cells following STRT1 knockdown. Western blot also demonstrated that SIRT1 depletion remarkably reduced the ratio of LC3-II/LC3-I and increased the protein level of p62 in H1299/CDDP cells (Fig. 4G), suggesting the inhibitory effect of si-STAT1 on cell autophagy in CDDP-resistant NSCLC cells. Moreover, SIRT1 silencing led to a remarkable decrease of FITC-LC3 positive dots in H1299/CDDP cells when compared to si-NC group (Fig. 4H), as evidenced by FITC-LC3 punctate formation analysis. Collectively, we concluded that SIRT1 silencing improved the chemosensitivity of CDDP-resistant NSCLC cells *via* repressing autophagy.

### miR-124 and miR-142 enhanced the chemosensitivity of CDDP-resistant NSCLC cells through repressing autophagy by targeting SIRT1

**Fig. 5:**
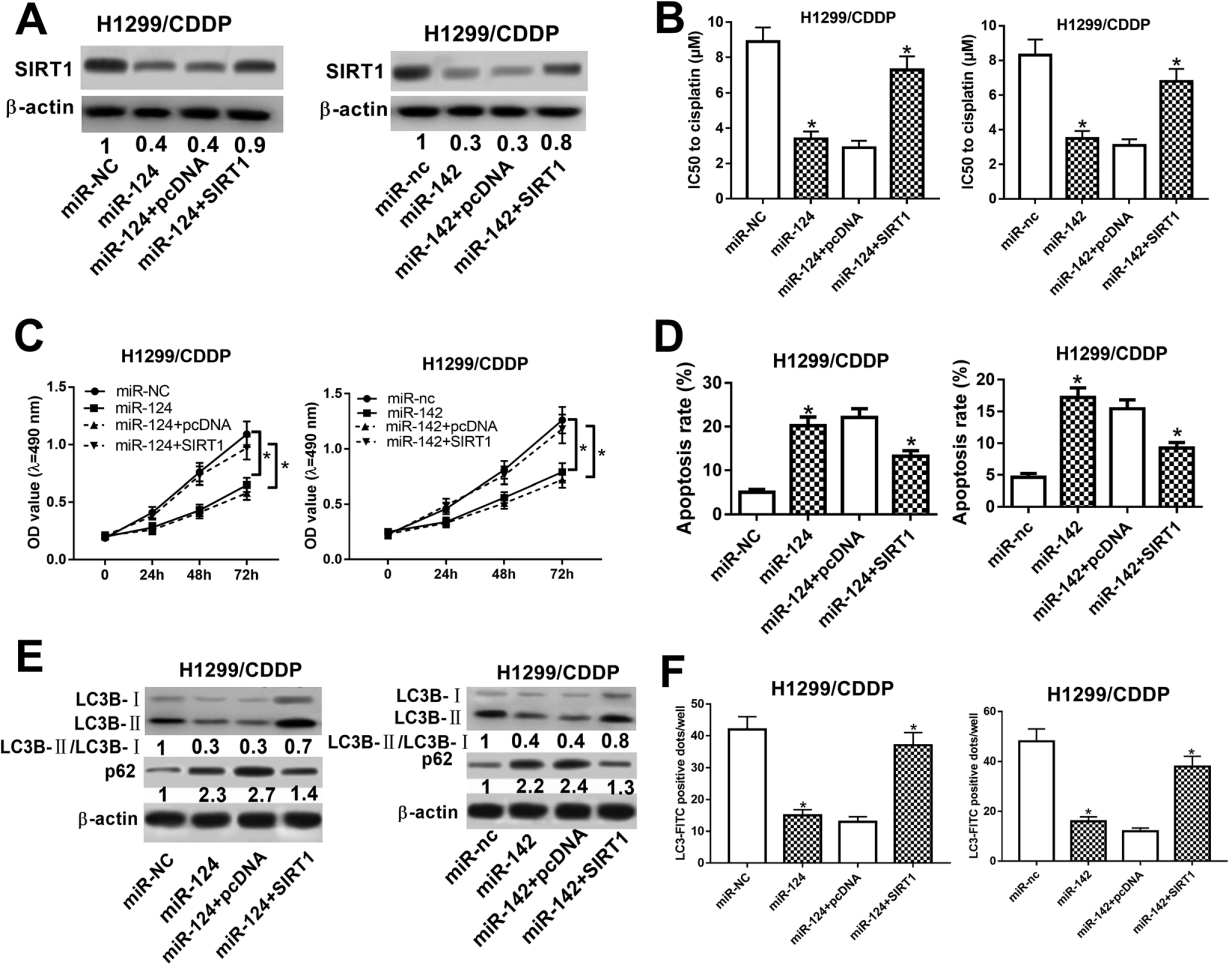
miR-124 and miR-142 inhibited autophagy and facilitated apoptosis in CDDP-resistant NSCLC cells by targeting SIRT1. H1299/CDDP cells were transfected with miR-124, miR-142, or matched controls or combined with SIRT1 or pcDNA. (A) The protein levels of SIRT1 in H1299/CDDP cells were detected by western blot assay. (B–D) MTT and flow cytometry assays were performed to measure the IC50 value, cell proliferation, and apoptosis. (E and F) Western blot and FITC-LC3 punctate formation were performed to assess cell autophagy. *P < 0.05.

To determine whether SIRT1 was a functional target of miR-124 and miR-142, we performed rescue experiments in miR-124 or miR-142-transfected H1299/CDDP cells through SIRT1 overexpression (Fig. 5A). MTT and flow cytometry analyses proved that exogenous SIRT1 effectively reversed miR-124 and miR-142-reduced IC50 value (Fig. 5B) and proliferation (Fig. 5C), as well as miR-124 and miR-142-induced apoptosis in H1299/CDDP cells (Fig. 5D). Additionally, western blot demonstrated that forced expression of miR-124 and miR-142 dramatically reduced the ratio of LC3-II/LC3-I and enhanced p62 level in H1299/CDDP cells, and the effects were evidently reversed in response to increased expression of SIRT1 (Fig. 5E). FITC-LC3 punctate formation analysis also manifested that miR-124 and miR-142-mediated decrease of FITC-LC3 positive cells was remarkably ameliorated following ectopic expression of SIRT1 in H1299/CDDP cells (Fig. 5F). These results indicated that SIRT1 overexpression overturned miR-124 and miR-142-mediated the chemosensitivity of CDDP-resistant NSCLC cells *via* repressing autophagy.

### miR-124 and miR-142p ameliorated the cisplatin sensitivity *in vivo*

**Fig. 6:**
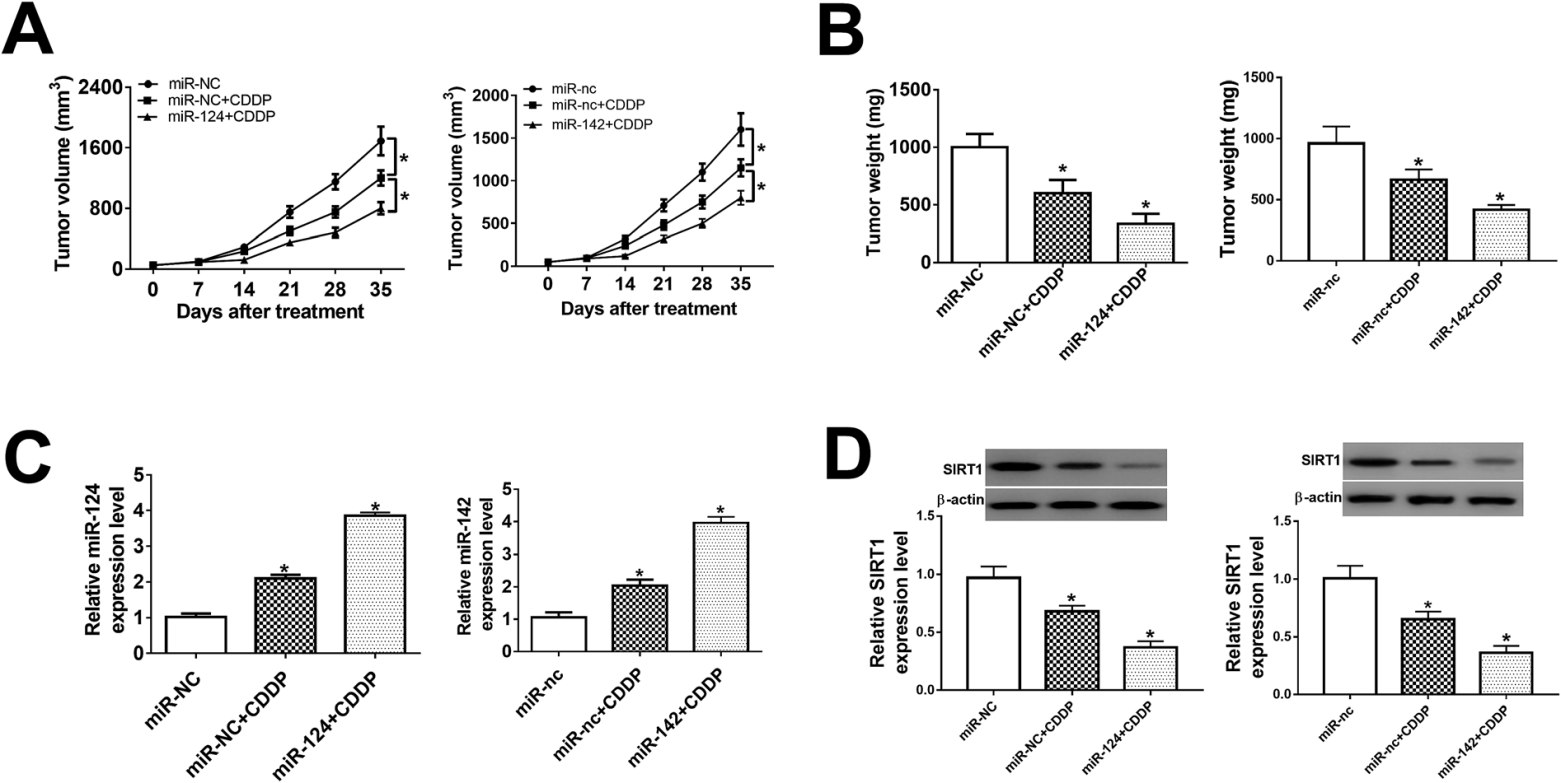
miR-124 and miR-142 enhanced chemosensitivity *in vivo*. BABL/c nude mice were injected subcutaneously with miR-NC, miR-124, miR-nc, or miR-142-transfected H1299/CDDP cells, and then treated with 10 mg kg^−1^ CDDP when tumor volume was about 60 mm^3^. (A) Tumor volume was measured at different periods after treatment. (B) Tumor weight was detected at end point. (C) miR-124 and miR-142 expressions in tumor tissues were detected by qRT-PCR. (D) The protein expression of SIRT1 in tumor was determined by western blot assay. *P < 0.05.

In present study, we further probe the effect of miR-124 and miR-142 on chemosensitivity *in vivo*. BABL/c nude mice were injected subcutaneously with miR-124, miR-142-transfected H1299/CDDP cells, followed by the treatment of 10 mg kg^−1^ CDDP when tumor volume was about 60 mm^3^. Results showed that tumor volume kept increasing in different groups until the end point (Fig. 6A). Overexpression of miR-124 or miR-142 suppressed tumor growth at each point after CDDP treatment compared with relevant control (Fig. 6A). Subsequently, tumor tissues were collected and weighted, showing that addition of miR-124 or miR-142 enhanced the inhibitory effect of CDDP on tumor growth (Fig. 6B). Furthermore, qRT-PCR and western blot assays were conducted to evaluate the expressions of miR-124, miR-142 and SIRT1 in each group. Introduction of miR-124 or miR-142 mimics significantly induced miR-124 or miR-142 expression (Fig. 6C), while suppressed SIRT1 protein expression (Fig. 6D). Taken together, upregulation of miR-124 or miR-142 improved the chemosensitivity of CDDP-resistant NSCLC to CDDP through negatively regulation of SIRT1 expression.

## Discussion

Although CDDP-based chemotherapy is extensively used in the treatment of NSCLC, resistance to CDDP-based chemotherapy is still the daunting challenge that limits its application.^21^ Evidence has been accumulating that dysregulation of miRNAs play important roles in the development of drug resistance in a wide range of malignancies, including NSCLC.^22^ For example, overexpression of miR-589, miR-1244, and miR-181b enhanced sensitivity to CDDP in NSCLC cells,^23,24^ while inhibition of miR-196a, and miR-10a reversed CDDP resistance.^25,26^ These results suggested the crucial roles of these miRNAs in the regulation of CDDP resistance in NSCLC. Hence, identification of tumor-related miRNAs and their target genes is of great significance for a thorough understanding of the mechanism underlying CDDP resistance and may provide novel therapeutic approaches for the chemotherapy of NSCLC.

In our study, we provided the first evidence that miR-124 and miR-142 were downregulated in CDDP-resistant NSCLC tumor tissues and cells, suggesting the potential association between miR-124, miR-142 and CDDP resistance in NSCLC. The role of miR-124 in drug resistance of various cancers is controversial.^27^ For example, Hu *et al.* revealed that miR-124 was downregulated in gefitinib-resistant NSCLC patients and cells and its overexpression sensitized gefitinib-resistant cells to gefitinib.^17^ In contrast, Liang *et al.* found that miR-124 was upregulated in prednisone insensitive acute lymphoblastic leukemia (ALL) cells and patients and contributed to glucocorticoid resistance of ALL cells.^28^ miR-142, located on human chromosome 17q22, has been reported to increase chemosensitivity of hepatocellular carcinoma,^29^ acute myelogenous leukemia,^30^ and NSCLC.^18^ Functionally, our study found that forced expression of miR-124 and miR-142 significantly suppressed autophagy and promoted apoptosis in CDDP-resistant NSCLC cells, which was consistent with the previous studies.^17,18^ To our knowledge, induction of autophagy by chemotherapeutic drugs helps cancer cells evade apoptosis, and thus contributes to the development of drug resistance in cancers.^31^ Based on these results, it is reasonable to confer that miR-124 and miR-142 reversed CDDP resistance in CDDP-resistant NSCLC cells by inhibition of autophagy. However, the underlying molecular mechanism by which miR-124 and miR-142 regulate autophagy of CDDP-resistant NSCLC cells is still far from being fully addressed.

SIRT1, a member of the sirtuin family, is a nicotinamide adenine dinucleotide (NAD^+^)-dependent histone deacetylase and closely associated with numerous biological processes, such as cell apoptosis, cell cycle, mammalian metabolism, aging, and cell survival.^32^ Tremendous studies have demonstrated that SIRT1 is frequently dysregulated in various tumors, and exerts oncogenic or tumor-suppressive roles in cancers, mainly depending on the specific cells or tumor types.^33,34^ In addition, it has been convincingly demonstrated that SIRT1 is closely related to cancer resistance to chemotherapeutic agents.^35^ For example, overexpression of SIRT1 was reported to be able to facilitate chemoresistance of ovarian cancer cells.^36^ Silencing of SIRT1 was shown to enhance the sensitivity to anti-cancer drugs in prostate cancer.^37^ In present study, we also demonstrated that diminished SIRT1 expression reversed CDDP resistance of NSCLC cells by inhibition of autophagy, which was fully consistent with previous studies.

In the following study, we aimed to explore whether miR-124 and miR-142 modulated chemosensitivity of CDDP-resistant NSCLC through suppressing autophagy *via* targeting the host gene. As a result, SIRT1 was considered to be a candidate target gene for miR-124 and miR-142. Afterwards, dual-luciferase report, RIP and western blot assays further demonstrated that miR-124 and miR-142 have the potential to interact with SIRT1 *via* the binding sites within SIRT1 3′UTR. During the last stage of our research, we revealed that SIRT1 upregulation overturned miR-124 and miR-142-enhanced CDDP sensitivity *via* modulation of cell autophagy in CDDP-resistant NSCLC cells.

All these data suggest that miR-124 and miR-142 enhanced the chemosensitivity of CDDP-resistant NSCLC cells through repressing autophagy by targeting SIRT1, which may provide a novel potential marker for gene treatment of CDDP-resistant NSCLC patients.

## Conflicts of interest

The authors declare none conflict of interest.

## Supplementary Material
